# Association between Elastic Modulus of Foot Soft Tissues and Gait Characteristics in Young Individuals with Flatfoot

**DOI:** 10.3390/bioengineering11070728

**Published:** 2024-07-18

**Authors:** Xin Jiao, Tianyi Hu, Yongjin Li, Binbin Wang, Mirabel Ewura Esi Acquah, Zengguang Wang, Qianqian Chen, Yaokai Gan, Dongyun Gu

**Affiliations:** 1 Shanghai Key Laboratory of Orthopedic Implant, Department of Orthopedic Surgery, Shanghai Ninth People’s Hospital, Shanghai Jiao Tong University School of Medicine, Shanghai 200011, China; jiaoxin2020@126.com (X.J.); wzg500189@163.com (Z.W.); ganyk2004@126.com (Y.G.); 2 School of Biomedical Engineering & Med-X Research Institute, Shanghai Jiao Tong University, Shanghai 200030, China; hutianyi0401@163.com (T.H.); stardust_lee@sjtu.edu.cn (Y.L.); wbb123@sjtu.edu.cn (B.W.); miry_a@sjtu.edu.cn (M.E.E.A.); 3 Engineering Research Center of Digital Medicine and Clinical Translation, Ministry of Education, Shanghai 200030, China; 4 Department of Ultrasound, Shanghai Ninth People’s Hospital, Shanghai Jiao Tong University School of Medicine, Shanghai 200011, China

**Keywords:** flatfoot, elastic modulus, gait, plantar fascia

## Abstract

Flatfoot is a common foot deformity, causing foot pain, osteoarthritis of the midfoot, and even knee and hip dysfunction. The elastic modulus of foot soft tissues and its association with gait biomechanics still remain unclear. For this study, we recruited 20 young individuals with flatfoot and 22 age-matched individuals with normal foot arches. The elastic modulus of foot soft tissues (posterior tibial tendon, flexor digitorum brevis, plantar fascia, heel fat pad) was obtained via ultrasound elastography. Gait data were acquired using an optical motion capture system. The association between elastic modulus and gait data was analyzed via correlation analysis. The elastic modulus of the plantar fascia (PF) in individuals with flatfoot was higher than that in individuals with normal foot arches. There was no significant difference in the elastic modulus of the posterior tibial tendon (PTT), the flexor digitorum brevis (FDB), or the heel fat pad (HFD), or the thickness of the PF, PTT, FDB, and HFD. Individuals with flatfoot showed greater motion of the hip and pelvis in the coronal plane, longer double-support phase time, and greater maximum hip adduction moment during walking. The elastic modulus of the PF in individuals with flatfoot was positively correlated with the maximum hip extension angle (r = 0.352, *p* = 0.033) and the maximum hip adduction moment (r = 0.429, *p* = 0.039). The plantar fascia is an important plantar structure in flatfoot. The alteration of the plantar fascia’s elastic modulus is likely a significant contributing factor to gait abnormalities in people with flatfoot. More attention should be given to the plantar fascia in the young population with flatfoot.

## 1. Introduction

Flatfoot, also called pes planus, refers to the condition where the curvature of the medial longitudinal arch is flatter than that of a healthy foot. The entire sole of the foot in individuals with flatfoot is in near-complete or complete contact with the ground [1]. The prevalence of flatfoot in young people is high, ranging from 5% to 57% depending on the age group [1,2,3]. A collapsed arch, consisting of the calcaneus, talus, navicular, three cuneiforms, and the first, second, and third metatarsals, is the main factor in flatfoot. As well as bony structures, the abnormal stiffness and stability of the soft tissues are the other causes of flatfoot [4,5,6,7]. Altered gait patterns leading to difficulties in walking are associated with flatfoot, which result in symptoms related to overuse injuries, midfoot arthritis, and hip and back pain [8,9,10]. However, the relationship between the properties of the soft tissues and the gait characteristics has not been reported. Understanding this relationship may contribute to developing corresponding treatments or preventions for flatfoot.

Foot soft tissues, such as tibialis posterior muscle, plantar aponeurosis, and spring ligament, play a key role in maintaining the medial longitudinal arch (MLA) [11]. Morphological changes in soft tissues, such as posterior tibial tendon, spring ligaments, deltoid ligaments, and abductor hallucis have been reported in individuals with flatfoot [12,13]. However, the alterations of some specific muscles are still controversial. The thickness of the flexor hallucis brevis was reported to be smaller in the study of Angin S et al. [14] but showed no significance in the study of Taş, S. et al. [13]. The mechanical properties of soft tissues have also attracted attention. These properties contribute to joint stability and control [15] and affect movement performance and rapid force production [16]. Studying the mechanical properties of plantar soft tissues helps in understanding the potential mechanisms of foot diseases and developing clinical treatments. Shear wave elastography (SWE) is a new development in soft tissues elasticity imaging. Unlike tensile or compression tests, the principle of SWE is that the safe acoustic radiation force from a linear-array US transducer generates the displacement of the particles in the tissue, which will produce the shear waves [17]. Young’s modulus (E) is defined as E = 3ρc_s_^2^, where ρ is the material density and c_s_ is the shear-wave velocity [18]. Through tracking and calculating the shear wave velocity, the Young’s modulus of the soft tissue will be obtained. The stiffness of the abductor hallucis muscle is greater in individuals with flatfoot compared to those with normal arches under high-loading conditions [19]. Additionally, the stiffness of the Achilles tendon is higher in the flatfoot group at rest but decreases during standing. Furthermore, the logarithmic decrement of the Achilles tendon is lower in individuals with flatfoot while standing [20]. Qian et al. reported that the thickness and Young’s modulus of the plantar fascia (PF) gradient decreased from the proximal to the distal end despite no comparison with the neutral arch [21]. These findings suggest the important role of alterations in the soft tissue biomechanical properties in flat feet. However, the mechanical properties of soft tissues are not yet fully understood.

Abnormal gait is another vital clinical symptom of flatfoot. Buldt et al. reported that in the transverse plane, individuals with flatfoot showed earlier peak abduction in the relationship between the rearfoot and leg and greater abduction at heel contact in the relationship between the lateral forefoot and the midfoot. In the frontal plane, individuals with flatfoot exhibited a reduced range of motion (ROM) in inversion/eversion in the relationship of midfoot and rearfoot. Additionally, people with flatfoot showed less peak plantarflexion during midstance in the sagittal plane in the relationship between the hallux and medial forefoot [22]. As well as foot joints, flatfoot is associated with proximal joint problems. Reductions in peak knee adduction moments during walking have been reported, which were shown to be correlated with lateral calcaneal shift [23]. Long-term abnormalities in gait pattern can lead to the development of osteoarthritis [24]. Investigating the mechanisms of gait disorders in flatfoot can help develop corresponding prevention or treatment strategies. However, the association between the elastic modulus of the foot soft tissues and gait characteristics in people with flatfoot remains unclear.

Therefore, in this study, we explored the association between the elastic modulus of plantar soft tissues and gait characteristics in individuals with flatfoot to deepen our understanding of gait abnormalities in individuals with flatfoot and identify potential therapies for the future.

## 2. Materials and Methods

### 2.1. Ethical Approval and Clinical Registration

This study was approved by the Ethics Committee of Shanghai Ninth People’s Hospital, Shanghai Jiao Tong University School of Medicine (Approval No.: SH9H-2023-T47-2), and was registered in the National Medical Research Registration and Archival Information System (https://www.medicalresearch.org.cn (accessed on 15 July 2024); number: MR-31-24-017699).

### 2.2. Participants Recruitment

A total of 20 individuals with flatfoot and 22 age-matched individuals with normal foot arches were recruited from Shanghai Ninth People’s Hospital, Shanghai Jiao Tong University School of Medicine. Each participant was informed of the full experimental process and possible risks and signed the informed consent forms. The inclusion criteria and exclusion criteria for participants with flat feet were as follows.

#### 2.2.1. Inclusion Criteria

(1) Diagnosed with flatfoot, and the diagnosis of flatfoot was confirmed by the same experienced surgeon from the Department of Foot and Ankle Surgery;
(2) Age 16–30 years old (each gender was permitted);
(3) No walking disorder;
(4) No language disorder.

#### 2.2.2. Exclusion Criteria

(1) Suffering from other orthopedic or neurological diseases, such as osteoarthritis of the knee;
(2) Suffering from severe osteoporosis or limited joint mobility;
(3) Suffering from previous stroke;
(4) Suffering from cognitive dysfunction;
(5) Suffering from lower extremity fracture or surgery of the lower extremity within 10 years;
(6) Suffering from malignant neoplastic disease either currently or within the previous 10 years (excluding non-melanoma skin cancer).

#### 2.2.3. Inclusion and Exclusion Criteria for Paticipants with Normal Foot Arches

The Inclusion Criteria and Exclusion Criteria for participants with normal foot arches were identical to participants with flat feet except the requirement of diagnosis with flatfoot

### 2.3. Ultrasound Data Acquisition

#### 2.3.1. Data Acquisition Process

**Figure 1:**
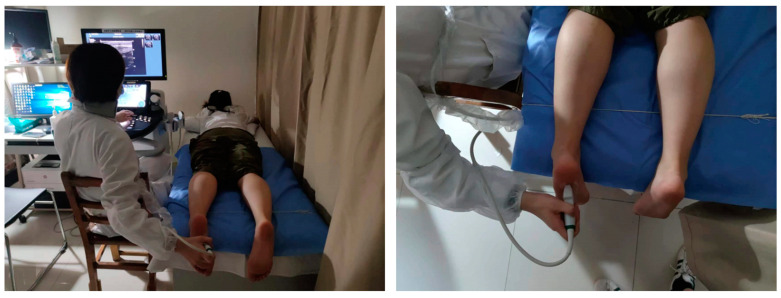
Acquisition of ultrasonic images.

Conventional B-mode ultrasound and SWE images were performed on the Aixplorer system (SuperSonic Imagine, Aix en Provence, France) by the same experienced sonographer from the Department of Ultrasound at Shanghai Ninth People’s Hospital, Shanghai Jiao Tong University School of Medicine. A 10-2 MHz linear-array transducer was employed. The participants were instructed to remove their shoes and socks and lay prone on the bed with their feet naturally relaxed and hanging off the edge of the examination bed (Figure 1). The selected soft tissues were scanned routinely. Once the images of the tissues were stable and clearly shown, their thickness was measured using B-mode ultrasound. Simultaneously, the SWE images were obtained without compression, with the selected soft tissues positioned at the center of the elasticity box. The images were saved after a few seconds to allow them to stabilize. Quantitative elasticity was measured in the region of interest (ROI). To assess intra-rater reliability, each elastic modulus measurement was taken once on three different days.

#### 2.3.2. Ultrasound Parameters

**Figure 2:**
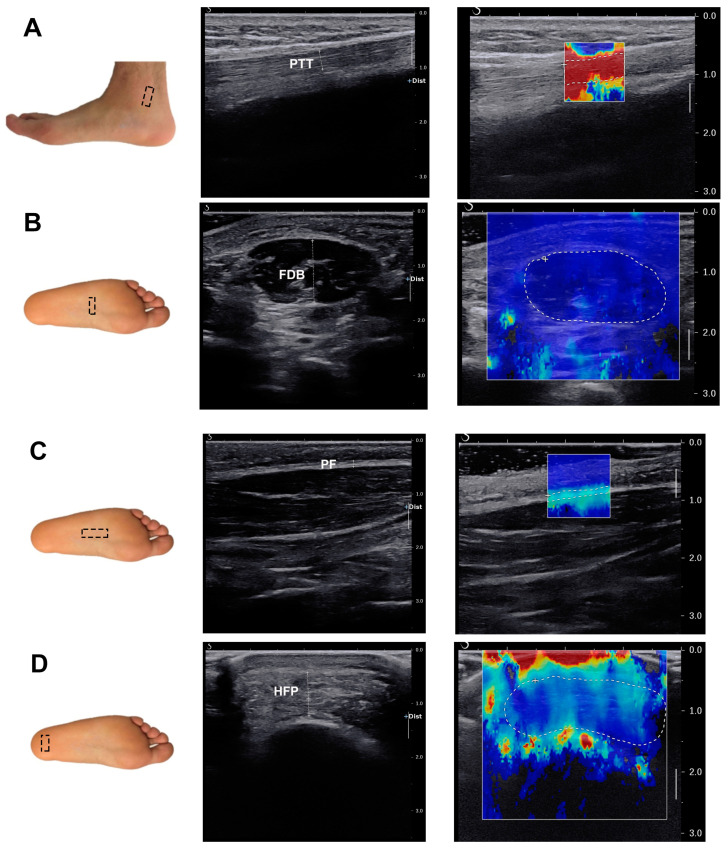
Probe location and ultrasound imaging measurements of PTT (**A**), FDB (**B**), PF (**C**), and HFP (**D**). The left column shows the locations of probes in the macro views. Dotted boxes represent the locations of the probe. The middle column displays the images of thickness of the four tissues. The right column exhibits the images of elastic modulus of the four tissues. Dashed zones represent ROIs of the four tissues. PTT: posterior tibial tendon; FDB: flexor digitorum brevis; PF: plantar fascia; HFP: heel fat pad.

Four specific tissues were measured, including the posterior tibial tendon (PTT), flexor digitorum brevis (FDB), plantar fascia (PF), and heel fat pad (HFD). The thickness of the PTT and PF was obtained from the long-axis gray-scale US image when they were showed clearly. In contrast, the thickness of FDB and HFD was measured on the short-axis US image at the maximum cross section of these two tissues. In SWE, the sampling frame was placed on the aforementioned soft tissues. A total of three stable SWE images at the same location were saved, and an ROI was drawn according to the boundary of the soft tissues to measure the Young’s modulus quantitatively on each image. The average elastic modulus from three measurements was used in this study. The locations of detection for these structures are shown in Figure 2.

### 2.4. Gait Data Acquisition

#### 2.4.1. Data Acquisition Process

The gait collection followed methods similar to those we have used previously [25]. Briefly, the gait analyses were performed by the same examiner at the Sino-UK Human Performance Laboratory, Shanghai Jiao Tong University. Twenty-two reflective markers in total were attached to the following anatomical positions of the participants: anterior superior iliac spine (ASIS); posterior superior iliac spine (PSIS); greater trochanter; medial and lateral femoral epicondyles; medial and lateral malleoli; posterior calcanei; and the head of the first, second, and fifth metatarsals. Additionally, four extra markers were placed on the lateral side of both shanks and thighs. A Vicon^®^ T40 motion capture system (Vicon Nexus 1.8.5, Oxford Metrics Ltd., Oxford, UK), operating at 100 Hz, was used to track the trajectory of these key points during the trials. Initially, the participants were asked to remain static for 3 s to construct static models. Then, participants were instructed to walk on a level 10 m walkway. Walking speed was controlled by the Timing Gait System (Brower Timing System, Draper, UT, USA). Participants were allowed to rest for at least 10 s between each successive walking trial. At least 20 trials were performed for each participant. Among these, trials with speeds within ±5% of the average speed were deemed successful. Six successful gait cycles from each participant were selected for analysis.

#### 2.4.2. Data Processing

Vicon Nexus 2.12 was initially employed to process the data. The kinematic data were first processed to fill any missing values using either spline fill or pattern fill methods. Then, the data were filtered using a second-order Butterworth low-pass filter with a cutoff frequency of 5 Hz. Finally, we obtained the kinematic data of each marker point in the x (coronal), y (sagittal), and z (vertical) axes. Next, the gait parameters were calculated with a commercially available software (Visual 3D, https://wiki.has-motion.com/Visual3D_Overview (accessed on 15 July 2024), C-motion Inc., Germantown, Maryland, USA). The gait cycles were defined according to gait events, namely, heel strike (HS) and toe off (TO), and normalized based on HS (0%) to TO (100%). After calibrating the gait events, the gait parameters were calculated by the software (Visual 3D).

#### 2.4.3. Gait Parameters

The spatiotemporal parameters included speed, stride width, step length, gait cycle, cadence, proportions of stance phase, swing phase, and double-stance phase.

The kinematics included maximum dorsal and plantar flexion, maximum internal and external rotation, and range of motion for the ankle joint; maximum flexion and extension, maximum adduction and abduction, maximum internal and external rotation, and range of motion for the knee joint; maximum flexion and extension, maximum adduction and abduction, maximum internal and external rotation, and range of motion for the hip joint; and maximum and minimum anterior tilt, maximum elevation and depression, maximum internal and external rotation, and range of motion for pelvis.

The kinetics included maximum dorsal and plantar flexion moment for the ankle joint; maximum flexion and extension moment and maximum adduction and abduction moment for the knee joint; and maximum flexion and extension moment and maximum adduction and abduction for the hip joint. All the net joint moments were external. The center of the hip joint was determined based on the method of Bell et al. [26]. The coordinates for the hip joint center along the x, y, and z axes were calculated as 0.36×ASIS_Distance, −0.19×ASIS_Distance, and −0.3×ASIS_Distance, respectively, where ASIS distance is the distance between the left and right anterior superior iliac spines. The center of the knee joint was determined by the geometric midpoint of the medial and lateral femoral condyles, and the center of the ankle joint was determined by the geometric midpoint of the medial and lateral malleoli.

### 2.5. Statistical Analysis

Quantitative parameters were expressed as mean ± standard deviation. Qualitative parameters were expressed as frequency. The Shapiro–Wilk test was performed for normality distribution. The intra-class correlation coefficient (ICC_1,1_) was used to assess the reliability of elastic modulus [19]. The independent sample Student’s *t* test was conducted on quantitative parameters. The chi-square test was conducted on qualitative parameters. Pearson’s r was calculated for correlation analysis. An r value greater than 0.8 was regarded as very strong, 0.6 to 0.8 as moderately strong, 0.3 to 0.5 as fair or moderate, and less than 0.3 as poor [27]. Statistical analyses were performed in SPSS, version 25.0 (IBM, Inc., Chicago, IL, USA). The level of statistical significance was set at *p* < 0.05.

## 3. Results

### 3.1. Baseline Characteristics of Participants

Twenty participants with flatfoot and twenty-two age-matched participants with normal foot arches (NF) were included in this study. As shown in Table 1, the demographics (age, gender, height and body mass) of the NF and flatfoot groups showed no significant difference (*p* > 0.05).

### 3.2. Reliability of Elastic Modulus Measurements

Shown in Table 2, the ICC was 0.995 for PTT, 0.960 for FDB, 0.967 for PF, and 0.973 for HFP. All these parameters exhibited near perfect reliability [28].

### 3.3. Thickness of Soft Tissues

Table 3 displays the thickness of the four soft tissues. The thickness of the four soft tissues did not show a significant difference between the two groups.

### 3.4. Elastic Modulus of Soft Tissues

Table 4 displays the elastic modulus of the four soft tissues. The elastic modulus of PTT, FDB, and HFD showed no significant difference between the two groups. Notably, the elastic modulus of PF in individuals with flatfoot is significantly higher than that of the NF group (*p* = 0.011), suggesting the important role of PF in flatfoot.

### 3.5. Gait Data

#### 3.5.1. Spatiotemporal Parameters

Table 5 exhibits gait spatiotemporal parameters in the two groups. No significant differences were found in speed, stride width, step length, gait cycle, cadence, stance phase, and swing phase. However, individuals with flatfoot showed a longer double-stance phase (0.092 ± 0.022%) than people with normal foot arches (0.083 ± 0.014%) (*p* = 0.005).

#### 3.5.2. Kinematics

In terms of kinematics, the flatfoot group showed a smaller maximum hip extension angle than individuals with normal foot arches (*p* = 0.002). Meanwhile, people with flatfoot exhibited a smaller minimum pelvis anterior tilt (*p* = 0.002) and a larger range of motion in pelvis tilt (*p* < 0.001) (Table 6).

#### 3.5.3. Kinetics

Individuals with flatfoot had a greater maximum hip adduction moment (*p* = 0.002). Individuals with flatfoot performed comparably to individuals with normal foot arches in other joint moment parameters and plantar reaction force parameters (Table 7).

### 3.6. Correlation Analysis

**Figure 3:**
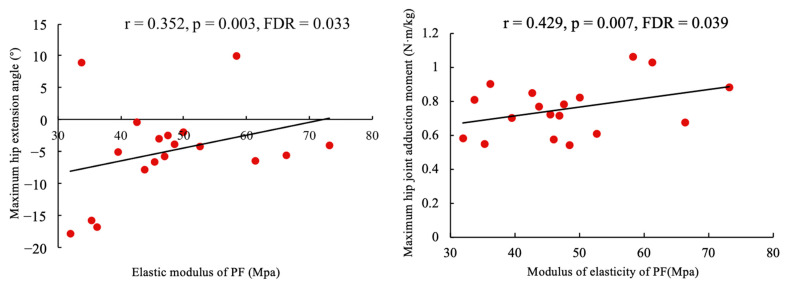
Correlation analysis between elastic modulus of soft tissue and gait features in participants with flatfoot.

The results of the correlation between soft tissue characteristics and gait biomechanical characteristics in individuals with flatfoot are shown in Figure 3. The maximum hip extension angle (r = 0.352, *p* = 0.003) and the maximum hip adduction moment (r = 0.429, *p* = 0.007) in individuals with flatfoot were positively correlated with the elastic modulus of PF.

## 4. Discussion

The role of soft tissues in the development of flat feet is not yet fully understood, making it essential to study their various characteristics. Reports on the mechanical properties of plantar soft tissues, particularly their association with abnormal gait, are still lacking. In this study, we examined the morphology and elastic modulus of plantar soft tissues in young participants with flatfoot. No significant differences were found in thickness between pes planus and normal feet under non-weight-bearing conditions. However, the elastic modulus of the plantar fascia was significantly higher in participants with flatfoot compared to individuals with normal foot arches. Additionally, we studied the gait patterns of participants with flatfoot. It was found that individuals with flatfoot showed a longer double-stance phase. They also displayed a smaller maximum hip extension angle, smaller minimum pelvis anterior tilt, and a larger range of motion in pelvis tilt. In terms of kinetics, participants with flatfoot showed a greater maximum of the hip adduction moment. Correlation analysis suggested the elastic modulus of PF was positively correlated with the maximum hip extension angle and the maximum hip adduction moment.

Previous studies have explored the morphology of soft tissues in flat feet. Angin S. compared the cross-sectional area and thickness of the vastus medialis and vastus lateralis muscles in normal feet and flat feet [14]. The thickness of the extensor hallucis longus, extensor hallucis brevis, and peroneus longus and peroneus brevis muscles was found to be significantly smaller in flat feet, whereas the extensor hallucis brevis muscles were significantly larger, reflecting compensatory muscular activity for reduced weight-bearing in individuals with flatfoot. Min Hwan Kim measured the thickness of the posterior tibialis brevis and peroneus longus muscles in children with flatfoot at the age of 4–8 and did not find any significant differences [29]. In our study, no significant soft tissue morphologic differences were found under non-weight-bearing conditions, indicating that the morphology of the soft tissues was not altered in essence and could return to normal from a weight-bearing to a non-weight-bearing condition in young individuals with flatfoot or early-stage flatfoot.

With respect to mechanical properties, we found that participants with flatfoot showed a higher elastic modulus of the plantar fascia (PF) compared to individuals with normal foot arches. The abnormal elastic deformation in the arch of the flat foot contributes to greater alternating stresses on the PF, leading to overuse injuries and impaired mobility function of the foot [30]. PF, located in the deeper part of the superficial plantar fascia, plays an important role in maintaining foot morphology and arch function. In detail, PF maintains the position of the heel bone and enhances the spring ligament’s support of the talar head, conducive to a more stable foot during the gait cycle [31,32]. A higher elastic modulus indicates more difficulty in deformation, suggesting that the PF of participants with flatfoot is less able to deform in a timely manner to adapt to different motion states.

As for the gait patterns, we found that the double support phase of gait was prolonged in participants with flatfoot. The double-support phase is the only phase of the entire gait cycle in which both feet are in contact with the ground, and it is also the shortest phase of the entire gait cycle in normal subjects. Many gait disorders are characterized by an extended double-support phase in the early stages in order to increase walking stability [33]. Dynamic instability in participants with flat feet has been reported previously [34]. In our study, the prolonged duration of the double-support phase may indicate balance problems in participants with flatfoot.

Apart from the longer double-support phase, we also found that individuals with flatfoot had smaller hip extension maxima and larger hip adduction moment maxima, together with smaller anterior pelvic tilt minima and larger anterior pelvic tilt ranges of motion, representing an increased level of hip and pelvic motion in the coronal plane during walking. These may represent compensation due to a collapsed foot arch. The main function of the hip joint is to provide dynamic support for the weight of the trunk, as well as to transfer loads between the axial skeleton and the lower limbs [35]. The pelvis tends to be more prone to abnormal muscle activation when subjected to abnormal joint angles [36]. Abnormal motion of the hip and pelvis is perhaps related to hip pain in flatfoot, as has been reported [37,38]. Thus, more attention should be paid to the hip and pelvis in individuals with flatfoot.

We also analyzed the correlation between the soft tissue characteristics and the gait features of the participants with flatfoot. We found that the maximum hip extension angle and the maximum hip adduction moment in these participants were positively correlated with the elastic modulus of the PF. This correlation likely arises because a greater elastic modulus of the plantar tendon membrane increases the stress on the PF, which impedes the adjustment of stress in the foot and leads to overall gait instability. Consequently, this instability requires an increase in the range of motion of the hip joint and an compensatory moment to maintain balance. Although the correlation is moderate, it still provides new insight into understanding the abnormal gait in flatfoot.

Our study had several limitations. First, our subjects were all young participants, which may not reflect the pathological conditions of middle-aged and older individuals with flatfoot. Second, the sample size was small, which may limit the generalizability of our findings. Third, we did not assess the regional heterogeneity of the soft tissue stiffness.

## 5. Conclusions

The plantar fascia is an important structure in the flat foot. The alteration of the elastic modulus of the plantar fascia is likely a significant contributing factor to gait abnormalities in individuals with flatfoot. More attention should be paid attention to the plantar fascia in the young population with flatfoot.

## Figures and Tables

**Table 1:** Baseline characteristics of participants.

	Age	Gender (Male/Female)	Height (cm)	Body Mass (kg)
Normal Foot (NF)	23.16 ± 3.90	10/12	167.28 ± 8.49	66.35 ± 13.30
Flatfoot	23.60 ± 1.56	10/10	167.87 ± 7.52	63.91 ± 12.70
*p*-value	0.600	0.768	0.819	0.558

**Table 2:** ICC of elastic modulus measurement via ultrasound.

	PTT	FDB	PF	HFP
ICC	0.995	0.960	0.967	0.973

**Table 3:** Thickness of foot soft tissues (cm).

	PTT	FDB	PF	HFD
NF	0.304 ± 0.026	0.955 ± 0.166	0.113 ± 0.012	0.846 ± 0.246
Flatfoot	0.293 ± 0.032	0.990 ± 0.206	0.115 ± 0.008	0.836 ± 0.178
*p*-value	0.081	0.397	0.276	0.832

**Table 4:** Elastic modulus of foot soft tissues (kPa).

	PTT	FDB	PF	HFD
NF	208.04 ± 30.374	13.002 ± 1.774	40.709 ± 9.742	18.821 ± 4.145
Flatfoot	197.56 ± 30.914	12.333 ± 2.515	46.730 ± 12.394	18.112 ± 3.978
*p*-value	0.117	0.165	0.011 *	0.421

* $P_{FDR}$ < 0.05.

**Table 5:** Gait spatiotemporal parameters.

	NF	Flatfoot	*p*-Value
Speed (m/s)	1.153 ± 0.080	1.184 ± 0.194	0.346
Stride width (m)	0.114 ± 0.026	0.118 ± 0.020	0.373
Step length (m)	0.618 ± 0.043	0.639 ± 0.064	0.084
Gait cycle (s)	0.536 ± 0.029	0.546 ± 0.046	0.255
Cadence (steps/min)	112.338 ± 6.084	110.806 ± 9.748	0.396
Stance phase (%)	0.619 ± 0.036	0.640 ± 0.065	0.073
Swing phase (%)	0.456 ± 0.028	0.456 ± 0.032	0.905
Double-stance phase (%)	0.083 ± 0.014	0.092 ± 0.022	0.005 *

* $P_{FDR}$ < 0.05.

**Table 6:** Kinematics (degrees).

Joint	Movement	Parameter	NF	Flatfoot	*p*
Ankle	Dorsi	Maximum dorsal	15.923 ± 2.713	15.869 ± 2.834	0.930
		Maximum plantar flexion	−13.903 ± 6.044	−15.061 ± 6.377	0.400
		range of motion	29.826 ± 4.807	30.93 ± 5.528	0.340
	Foot Progression Angle	Maximum toe-in	−7.925 ± 4.970	−9.198 ± 7.451	0.371
		Maximum toe-out	−23.238 ± 5.536	−23.689 ± 7.924	0.768
		range of motion	15.313 ± 3.916	14.491 ± 4.594	0.388
Knee	Flexion	Maximum flexion	66.397 ± 5.997	66.561 ± 5.240	0.895
		Maximum extension	4.414 ± 4.126	4.786 ± 5.155	0.722
		range of motion	61.983 ± 5.002	61.775 ± 2.935	0.817
	Adduction	Maximum adduction	6.992 ± 4.488	6.375 ± 4.967	0.558
		Maximum abduction	−5.225 ± 4.793	−5.074 ± 3.359	0.869
		range of motion	12.217 ± 4.050	11.45 ± 3.808	0.379
	Rotation	Maximum internal rotation	−0.131 ± 5.981	1.238 ± 4.848	0.256
		Maximum external rotation	−17.6 ± 6.340	−15.631 ± 5.596	0.139
		range of motion	17.469 ± 5.714	16.869 ± 3.425	0.562
Hip	Flexion	Maximum flexion	38.112 ± 8.991	35.331 ± 7.496	0.131
		Maximum extension	0.479 ± 10.313	−4.149 ± 7.502	0.002 *
		range of motion	37.633 ± 4.774	39.48 ± 4.760	0.084
	Adduction	Maximum adduction	7.230 ± 3.850	7.018 ± 3.410	0.792
		Maximum abduction	−5.995 ± 3.462	−6.403 ± 3.560	0.601
		range of motion	13.224 ± 3.240	13.42 ± 3.433	0.792
	Rotation	Maximum internal rotation	6.922 ± 11.023	6.185 ± 9.952	0.750
		Maximum external rotation	−6.445 ± 10.166	−7.043 ± 8.508	0.773
		range of motion	13.368 ± 3.960	13.228 ± 3.489	0.866
Pelvis	Tilt	Maximum anterior tilt	14.744 ± 7.530	11.057 ± 5.289	0.012
		Minimum anterior tilt	12.032 ± 7.656	7.540 ± 5.156	0.002 *
		range of motion	2.712 ± 0.622	3.517 ± 1.069	<0.001 *
	Obliquity	Maximum elevation	3.938 ± 2.495	3.555 ± 2.204	0.463
		Maximum depression	−4.051 ± 2.519	−3.578 ± 2.213	0.369
		range of motion	7.989 ± 2.861	7.134 ± 1.923	0.113
	Rotation	Maximum internal rotation	7.198 ± 2.994	7.34 ± 3.534	0.845
		Maximum external rotation	−6.194 ± 3.226	−7.082 ± 3.205	0.216
		range of motion	13.392 ± 4.228	14.422 ± 4.386	0.283

* $P_{FDR}$ < 0.05; negative joint angles represent plantar flexion of ankle, toe-out-of-foot progression angle, extension, abduction, external rotation of knee, extension, abduction, external rotation of hip, posterior tilt, depression, and external rotation of pelvis.

**Table 7:** Kinetics (N·m/kg).

Joint	Movement	NF	Flatfoot	*p*
Ankle	Maximum dorsal	1.367 ± 0.122	1.496 ± 0.179	0.324
	Maximum plantar flexion	−0.161 ± 0.080	−0.181 ± 0.100	0.320
Knee	Maximum flexion	0.709 ± 0.185	0.729 ± 0.207	0.277
	Maximum extension	−0.285 ± 0.046	−0.264 ± 0.099	0.221
	Maximum adduction	0.402 ± 0.125	0.436 ± 0.225	0.325
	Maximum abduction	−0.116 ± 0.044	−0.092 ± 0.079	0.106
Hip	Maximum flexion	0.618 ± 0.157	0.659 ± 0.188	0.318
	Maximum extension	−0.650 ± 0.140	−0.700 ± 0.340	0.570
	Maximum adduction	0.770 ± 0.080	0.880 ± 0.080	0.002 *
	Maximum abduction	−0.180 ± 0.050	−0.160 ± 0.120	0.640

* $P_{FDR}$ < 0.05. Negative joint angles represent plantar flexion moment of ankle, extension, abduction moments of knee, extension, and abduction moments of hip.

## Data Availability

The data presented in this study are available on request from the corresponding author.
